# Supplementary material for: Impact assessment for just transition of protein production systems
Source: PLoS One. 2025 Aug 1;20(8):e0328789. doi: 10.1371/journal.pone.0328789 (PMC12316291; doi:10.1371/journal.pone.0328789)
Supplement: S1 Table — (DOCX) [file pone.0328789.s001.docx]

**S1 Table. Protein production systems and their descriptions.**

|  | **Protein type** | **Protein production system** | **Management system** | **Definition** |
| --- | --- | --- | --- | --- |
| 1 | Beef | Traditional beef | Pastoralist, Tanzania and Kenya | Pastoralists, such as the Masai, are a semi-nomadic community that migrates within semi-arid lowlands and more humid uplands to obtain water and pasture. Beef production is a subsistence farming activity for the majority of the population and provides localised food security. Cattle play a key cultural role in ceremony, hierarchy, economic security, and determine social roles around milking, grazing and other aspects of pastoralism [1-4]. |
| 2 |  | Industrial scale beef | Small-scale, China | Small-scale concentrated cattle production systems that are dependent on animal feed, grains, and crop residues and comprise of 8-10 cattle heads per farm. Such small-scale intensive beef systems represent more than 50% of the beef industry in China. Such farms also include slaughtering of animals [1,5-7]). |
| 3 |  |  | Mixed grains and livestock, UK | Mixed farming involves running a system of livestock and arable crops on the same farm and traditionally involves a broad range of crops and livestock being grown and raised. This spreads the risk across a number of different crops. Much of the livestock feed is grown on farms, and livestock manure is used for crop fertiliser. Mixed farms typically have natural field margins and hedgerows, providing a range of habitats that support biodiversity [1,5,8-10]. |
| 4 |  |  | Organic, Europe | Organic beef systems are typically mixed crop and livestock operations without the use of synthetic chemicals. Cattle are often organic grain fed or organic grass fed or a combination of both. Crops and pastures are rotated and rested to replenish soils, and to control animal parasites [1,8,9,11]. |
| 5 |  |  | Confined feeding operations, US | A Confined Animal Feeding Operation (CAFO) is defined as a large animal feeding operation with more than 1000 cattle confined for over 45 days a year. CAFO are highly industrial beef production systems where cattle are fed concentrated animal feed throughout the year. Animal manure and wastewater are usually discharged into a natural or man-made ditch, stream or other waterways. They are the dominant animal production industry in the USA [1,8,9]. |
| 6 |  | Extensive beef | Rangelands, Australia | Rangelands include an extensive region of low rainfall with arid and semi-arid climates, and also some tropical and sub-tropical climates in far north Australia. Cattle graze on native herbaceous or shrubby vegetation [8,9,12]. |
| 7 |  |  | Regenerative, Global | Regenerative beef production includes rotational grazing and improving soil health. It involves allowing animals to graze on (and eat) real grass with rich soil, which contributes more nutrients to plants and grasses, resulting in more nutritious meat from the animals [13,14]. |
| 8 | Pork | Pork | Industrial pork, China | Small-scale intensive pig production systems account for over half of the total number of pig farms in China. Almost 98 per cent of the total pork production is by small-scale farmers who have fewer than 100 pigs. They are fed with globally sourced oilseeds and grains feed, antibiotics and other growth promoters commonly used [15-17]. |
| 9 | Poultry | Poultry | Caged poultry, India | Cage poultry includes raising hens in confined spaces where they are unable to roam and explore. It is a highly intensive system providing less than one square foot of area per bird. They are fed with concentrated grains and feed, and antibiotics and growth regulators are also commonly used [1,18,19]. |
| 10 |  |  | Free-range poultry, Europe | Free-range poultry farming includes access to open pastures during daytime. Up to 10000 birds per hectare can be housed. They still require concentrated feed, and antibiotics [1,20,21]. |
| 11 | Plant-based | Plant-based | Plant-based, Global | Plant protein is a food source of protein that comes from plants. This group can include pulses, tofu, soya, nuts, seeds, certain grains and even peas. Production of these crops includes intensive farming with high input of agrochemicals, heavy machinery, and high energy use on the farm. Plant-based alternative food involves much processing, which requires high energy. Here we include plant-based alternative food and not the direct consumption of a variety of plant-based proteins (pulses, nuts, seeds, peas and beans) [22-24]. |
| 12 | Cultivated meat | Cultivated meat | Conventional energy, Cultivated meat, Global | Cultivated meat is produced by cultivating animal cells directly in bioreactors using conventional energy. This production method eliminates the need to raise and farm animals for food [25-28]. |
| 13 |  |  | Sustainable energy, Cultivated meat, Global | Cultivated meat is produced by cultivating animal cells directly in bioreactors using renewable energy. This production method eliminates the need to raise and farm animals for food [25-28]. |

**References used in the above table**

1. Steinfeld H, Mäki-Hokkonen J. A classification of livestock production systems. World. Anim. Rev. 1995; 83-94. <https://www.fao.org/3/v8180t/v8180t0y.htm>
2. Mapinduzi AL, Oba G, Weladji RB, Colman JE. Use of indigenous ecological knowledge of the Maasai pastoralists for assessing rangeland biodiversity in Tanzania. Afri. J. Ecol. 2003; 41(4): 329-336.
3. Roba HG, Oba G. Integration of herder knowledge and ecological methods for land degradation assessment around sedentary settlements in a sub-humid zone in northern Kenya. Int. J. Sustain. Dev. World. Ecol. 2008; 15(3): 251-264.
4. Robinson TP, Thornton PK, Francesconi GN, Kruska R, Chiozza F, Notenbaert AMO, Cecchi G, Herrero MT, Epprecht M, Fritz S. Global livestock production systems. FAO and ILRI. 2011. Available from: <https://www.fao.org/3/I2414E/i2414e.pdf>
5. De Wit J, Westra PT, Nell AJ. Environmental Impact Assessment of Landless Livestock Ruminant Production System. Livestock, Environment and Development (LEAD) Initiative. Food and Agriculture Organization of United Nations and World Bank. 1996. Available from: <https://www.fao.org/3/X6111E/x6111e00.htm#Contents>
6. Wei S, Bai ZH, Chadwick D, Hou Y, Qin W, Zhao ZQ, Jiang RF, Ma L. Greenhouse gas and ammonia emissions and mitigation options from livestock production in peri-urban agriculture: Beijing–A case study. J. Clean. Prod. 2018; 178: 515-525.
7. Greenwood PL. Review: An overview of beef production from pasture and feedlot globally, as demand for beef and the need for sustainable practices increase. Animal. 2021; 15: 100295. <https://doi.org/10.1016/j.animal.2021.100295>
8. Sandhu HS, Wratten SD, Cullen R, Case B. The future of farming: the value of ecosystem services in conventional and organic arable land. An experimental approach. Ecol. Econ. 2008; 64: 835-848.
9. Sandhu H. Bottom-up transformation of agriculture and food systems. Sustainability. 2021; 13(4): 2171.
10. Mayerfeld D, Keeley KO, Rickenbach M, Rissman A, Ventura SJ. Evolving conceptions of silvopasture among farmers and natural resource professionals in Wisconsin, USA. Front. Sustain. Food. Syst. 2023; 7: 983376. <https://doi.org/10.3389/fsufs.2023.983376>
11. Pishgar-Komleh SH, Beldman A. Literature review of beef production systems in Europe. Wageningen Livestock Research. 2022. Report 2022. 39 pp. Available from: <https://edepot.wur.nl/567148>
12. Bentley D, Hegarty RS, Alford AR. Managing livestock enterprises in Australia's extensive rangelands for greenhouse gas and environmental outcomes: a pastoral company perspective. Aust. J. Exp. Agric. 2008; 48(1-2): 60-64.
13. Newton P, Civita N, Frankel-Goldwater L, Bartel K, Johns C. What is regenerative agriculture? A review of scholar and practitioner definitions based on processes and outcomes. Front. Sustain. Food. Syst. 2020; 4: 577723. <https://doi.org/10.3389/fsufs.2020.577723>
14. Schulte LA, Dale BE, Bozzetto S, Liebman M, Souza GM, Haddad N et al. Meeting global challenges with regenerative agriculture producing food and energy. Nat. Sustain. 2022; 5(5): 384-388.
15. Lander B, Schneider M, Brunson K. A history of pigs in China: from curious omnivores to industrial pork. J. Asian. Stud. 2020; 79(4): 865-889.
16. Robinson TP, Thornton PK, Franceschini G, Kruska RL, Chiozza F, Notenbaert A. et al. Global livestock production systems. Rome, Food and Agriculture Organization of the United Nations (FAO) and International Livestock Research Institute (ILRI), 152 pp. 2011. Available from: <https://www.fao.org/4/i2414e/i2414e.pdf>
17. Dai XW, Sun Z, Muller D. Driving factors of direct greenhouse gas emissions from China's pig industry from 1976 to 2016. J. Integr. Agric. 2021; 20(1): 319-329.
18. Baruah MS, Bhatt BP. Recycling of caged layer manure as broiler feed. Indian. Vet. J. 2008; 85(3): 293.
19. Singh R. Status of poultry production in India. 2019. Available from: <https://www.pashudhanpraharee.com/status-of-poultry-production-in-india/>
20. Taylor RC, Omed H, Edwards-Jones G. The greenhouse emissions footprint of free-range eggs. Poult. Sci. 2014; 93(1): 231-237.
21. Vaarst M, Steenfeldt S, Horsted K. Sustainable development perspectives of poultry production. World. Poult. Sci. J. 2015; 71(4): 609-620.
22. de Boer J, Aiking H. On the merits of plant-based proteins for global food security: Marrying macro and micro perspectives. Ecol. Econ. 2011; 70(7): 1259-1265.
23. Goldstein B, Moses R, Sammons N, Birkved M. Potential to curb the environmental burdens of American beef consumption using a novel plant-based beef substitute. Plos One. 2017; 12(12): e0189029. <https://doi.org/10.1371/journal.pone.0189029>
24. Sadhukhan J, Dugmore TIJ, Matharu A, Martinez-Hernandez E, Aburto J, et al. Perspectives on "game changer" global challenges for sustainable 21st century: plant-based diet, unavoidable food waste biorefining, and circular economy. Sustainability. 2020; 12(5): 1976. <https://doi.org/10.3390/su12051976>
25. Sharma S, Thind SS, Kaur A. In vitro meat production system: why and how? J. Food. Sci. Technol. 2015; 52(12): 7599-7607.
26. Bhat ZF, Morton JD, Mason SL, Bekhit AEDA, Bhat HF. Technological, regulatory, and ethical aspects of in vitro meat: A future slaughter‐free harvest. Compr. Rev. Food. Sci. Food. Saf. 2019; 18(4): 1192-1208.
27. Newton P, Blaustein-Rejto D. Social and economic opportunities and challenges of plant-based and cultured meat for rural producers in the US. Front. Sustain. Food. Syst. 2021; 5: 624270.
28. Sinke P, Swartz E, Sanctorum H, van der Giesen C, Odegard I. Ex‑ante life cycle assessment of commercial‑scale cultivated meat production in 2030. Int. J. Life Cycle Assess. 2023; 28: 234–254. <https://doi.org/10.1007/s11367-022-02128-8>
