# Supplementary material for: Impact assessment for just transition of protein production systems
Source: PLoS One. 2025 Aug 1;20(8):e0328789. doi: 10.1371/journal.pone.0328789 (PMC12316291; doi:10.1371/journal.pone.0328789)
Supplement: S2 Table — (DOCX) [file pone.0328789.s002.docx]

**S2 Table. Impact categories and their definitions.**

|  | **Categories** | **Definitions** |
| --- | --- | --- |
|  | **Natural capital** | |
| 1 | Net GHG emissions | Greenhouse gas (GHG) emissions produced by livestock (methane and nitrous oxide), crop production (livestock feed and plant-based protein base material), farm activities (machinery, animal housing, etc.), production facilities (plant-based), transport (to farm, not to market), meat/protein processing and packaging. It includes any carbon sequestration in the production systems. |
| 2 | Freshwater extraction and water pollution | Freshwater used to feed livestock, water crops, process meat/protein, clean and operate facilities and machinery. Also includes water pollution from protein production (chemicals, medication, manure). |
| 3 | Waste generation (on farm) | Waste produced throughout the protein production process (manure, litter, chemical waste, processing waste). |
| 4 | Air pollution | Methane, nitrous oxide, odour (manure – hydrogen sulphide, ammonia), organic dust (feed components, dried faecal matter, dander, mould, pollen, grains, mites, insect parts, litter and feathers; dust carries bacteria and viruses) |
| 5 | Loss of livestock biodiversity | The loss of livestock diversity by only using selected breeds, or crops or cells (cultured meat). |
| 6 | Loss of agri-enviro biodiversity (on farm) | The impact of protein production on the flora and fauna (insects, birds, animals, soil microbes) that exist in the landscape in addition to the livestock and crops. This includes green margins, fallow areas on the farm. |
| 7 | Land use change (indirect) | Change in land use to produce crops to supply (input) protein production systems. For example, growing crops for animal feed or plant matter for plant-based protein. |
| 8 | Land degradation (direct) | Degradation of the land caused by protein production systems. For example, deforestation to enable grazing. |
| 9 | Soil contamination | Contamination of soils from the use of agrochemicals, organic pollutants, soil erosion etc. |
|  | **Human** | |
| 10 | Skills and knowledge of workers, training | Formal education, and informal – learning from colleagues/family/network, skills/knowledge required to do the job. |
| 11 | Workplace health and safety standards | Regulated industries, better conditions, deregulated generally poorer conditions, but small systems can be better with appropriate standards for health and safety. |
| 12 | Health of farmers | Physical and mental health from working seven days a week, lifestyle of farmers etc. |
| 13 | Health of farm and production workers | Health and wellbeing of farm workers. |
| 14 | Consumers health | Risk for disease from production processes (antibiotics, chemicals, hormones), and from eating different types of protein (processed red meat, plant-based). |
|  | **Social** | |
| 15 | Community engagement | Engagement of farmers with the local community. |
| 16 | Networks | Industry networks, for learning, sharing, collaborating. |
| 17 | Employment | Employment generation on the farm. |
| 18 | Livelihood of livestock farmers | Opportunities for the livelihood of farmers - meaningful work, income, and wellbeing. |
| 19 | Opportunities for young people | Employment, training, and future prospects of the young generation to be involved in farming. |
|  | **Produced** | |
| 20 | Energy use | Use of electricity, gas, diesel on the farm, for housing animals, farm machinery, heating, cooling and transport |
| 21 | Animal health | Healthy and happy animals grazing in the open. Animal treatment and welfare on farm. |
| 22 | Productivity | Geographical area and production per unit area per unit time. |
| 23 | Profits | Return on investment – after expenses. |
|  | **Governance** | |
| 24 | Influence on policies and regulations | Lobbying power by farmers to change legislation, policy. |
| 25 | Governance structure | People, rules, policy, decision making structure at the farm level. |

Note: An extensive list of references for each of the above indicators is provided in the Excel Database.
