# Supplementary material for: Impact assessment for just transition of protein production systems
Source: PLoS One. 2025 Aug 1;20(8):e0328789. doi: 10.1371/journal.pone.0328789 (PMC12316291; doi:10.1371/journal.pone.0328789)
Supplement: S4 Table — (DOCX) [file pone.0328789.s004.docx]

**S4 Table. A 10-point impact scale used in this study.**

| **Scale** | **Impact** |
| --- | --- |
| -5 | extreme negative impact |
| -4 | very high negative impact |
| -3 | high negative impact |
| -2 | medium negative impact |
| -1 | low negative impact |
| 1 | low positive impact |
| 2 | medium positive impact |
| 3 | high positive impact |
| 4 | very high positive impact |
| 5 | extreme positive impact |

0: No information, NA: Not applicable
