# Supplementary material for: Impact assessment for just transition of protein production systems
Source: PLoS One. 2025 Aug 1;20(8):e0328789. doi: 10.1371/journal.pone.0328789 (PMC12316291; doi:10.1371/journal.pone.0328789)
Supplement: Attachment — Submitted filename: Response to reviewers.docx [file pone.0328789.s005.docx]

**PONE-D-25-10342**

**Editor’s comments**

Thank you for giving us the opportunity to revise our manuscript. We have addressed both the editor’s and reviewers’ comments below. Please refer to the Word file, Revised manuscript with track changes, for the changes mentioned below.

|  | **Comment** | **Response** |
| --- | --- | --- |
| 1 | Format the manuscript using the PLOS ONE style | Thanks for this comment. We have now carefully formatted the manuscript according to PLOS ONE's style requirements. |
| 2 | Financial disclosure | We have provided this statement in the cover letter.  The authors would like to acknowledge the financial support provided by the Federation University Australia and Aleph Farms joint grant G2355. Priyambada Joshi (PhD candidate) received stipend/financial support from this joint research project funded by Federation University Australia and Aleph Farms. Lee Recht reports a relationship with Aleph Farms that includes employment. Co-author Dr Lee Recht was previously employed at Aleph Farms during the data analysis and writing the first draft of the article. Dr Recht has no longer worked at this organisation since August 2024. Other authors declare that they have no known competing financial interests or personal relationships that could have appeared to influence the work reported in this paper. |
| 3 | Include full ethics statement in the Methods section. | Thanks for this comment. We have included it in the Methods section page 10, Delphi approach section. Lines 275-280. |
| 4 | Review reference list | We have carefully checked all references cited in the paper and in the list. We have also revised the style of all references provided in the Supplementary Information and the Excel Database. |

**Response to reviewers’ comments**

**Reviewer 1**

|  | **Comment** | **Response** |
| --- | --- | --- |
| 1 | The manuscript is technically sound, has sufficient sample sizes, controls and replicates that have undergone statistical processing. The work is presented using a large amount of data indicating the relevance of the development. | Thank you for your comment and encouragement. |
| 2 | Due to the fact that the relevance of this problem remains for a long time, for the appropriate conclusions, it is advisable to refer to modern works touching on similar topics, for example, in the following publications: DOI 10.1163/23524588-20230025; Development of a technology for the isolation and purification of insectoprotein from Zophobas morio biomass / I. V. Rzhepakovsky, P. A. Trushov, S. N. Povetkin, A. A. Naghdalyan // Modern science and innovation. - 2018. - No. 2 (22). - P. 85-91. - EDN VMYUHK.; Zophobas Morio Semi Industrial Cultivation Peculiarities / A. A. Nagdalian, S. V. Pushkin, I. V. Rzhepakovsky [et al.] //  Entomology and Applied Science Letters. – 2018. – Vol. 5, No. 2. – P. 108-113. – EDN WFWHYJ.; Why does the protein turn black while extracting it from insects biomass? / A. A. Nagdalian, N. P. Oboturova, S. N. Povetkin [et al.] //  Journal of Hygienic Engineering and Design. – 2019. – Vol. 29. – P. 145-150. – EDN POKELO.; [https://doi.org/10.1051/e3sconf/202453703007](https://url.au.m.mimecastprotect.com/s/V-o8Cp81w3uDVQyqsVSGfGWP28?domain=doi.org). | Thank you for these helpful suggestions to include work on insect proteins. We have included them in the discussion section, limitations on page 20, lines 578-579. References 60-62.  Rzhepakovskiy IV, Trushov PA, Povetkin SN, Nagdalian AA. 2018. The development of technology of protein extraction and purification in *Zophobas Morio*. Modern Science and Innovations 2, 85-91. (In Russ.)  Nagdalian AA, Pushkin SV, Rzhepakovsky IV, Povetkin SN, Simonov A N, Verevkina MN, Ziruk IV. 2019. *Zophobas Morio* semi industrial cultivation peculiarities. Entomology and Applied Science Letters 5, 108-113  Nagdalian AA, Oboturova NP, Krivenko DV, Povetkin SN, Blinov AV, Verevkina MN, Marinicheva MP, Simonov AN, Rodin IA, Lakovets MG. 2019. Why does the protein turn black while extracting it from insect’s biomass? Journal of Hygienic Engineering and Design 29, 145-150. |
| 3 | The text should be carefully checked for typos and grammatical errors. | Thank you for this comment. The entire manuscript and the supplementary information have been proofread by a professional copy editor. |

**Reviewer 2**

|  | **Comment** | **Response** |
| --- | --- | --- |
| 1 | The manuscript must include the recent research works on  application to protein production systems, especially from the past 5 years | We thank the reviewer for this comment. As stated in the manuscript's methods section, we conducted a thorough systematic review of studies published until January 2025, including studies from 1986 onwards. In addition to these studies, we have included some recently published work on protein production in the introduction and conclusion to further strengthen our arguments.  See below for details on the various recent references being added to support our arguments.  Introduction: References 9,10,13,18  Discussion: References 46,51,60-62 |
| 2 | Introduction Section-Authors should justify here why this study is important and should state the uniqueness of this study. | Thank you for this helpful comment. We now clearly describe the importance of the study and state its uniqueness in the introduction section (see page 3, lines 50-57). We have also included an updated reference to support this (13). |
| 3 | Please include the recent references for broad readers to understand the importance of protein production from various sources and in the industrial applications. | Thank you for your suggestion. The paper focuses on livestock-based protein production systems, with two protein innovations (plant-based and cultivated meat). However, as per the suggestions, we have included similar references from various sources, including industrial applications.  See new references, 9,10, 13,18,46,51, 60-62. |
| 4 | There are numerous grammatical errors and they significantly affect the flow of ideas. Please check through the MS. | Thank you for this comment. A professional copy editor has proofread the entire manuscript and the supplementary information. |
| 5 | Please add line numbers for easy identification of specific lines in the manuscript during review. | We have added line numbers to the manuscript. |
| 6 | Research would be nice if you could include the latest updates on recent studies. | Thank you for your suggestion. We have included the following references in the introduction and discussion sections to support our statements.  See new references, 9,10, 13,18,46,51, 60-62. |
| 7 | Check thoroughly all typographical errors and references, and the action should follow the journal guidelines. | Thank you for this comment. A professional copy editor has proofread the entire manuscript and the supplementary information. |
| 8 | Conclusions need to be elaborated and made clearer. | Thank you for this suggestion. We have now added two main conclusions from our findings in the conclusion section.  See page 21, lines 604–614. |
